# Age Structure of Water Frogs of the Genus *Pelophylax* in the Middle Volga River Region (European Russia)

**DOI:** 10.3390/ani15091273

**Published:** 2025-04-30

**Authors:** Renat Zamaletdinov, Anton Svinin, Alexander Fayzulin, Oleg Ermakov, Regina Mikhaylova, Spartak Litvinchuk

**Affiliations:** 1 Department of Environmental Engineering and Water Resources Management, Kazan (Volga Region) Federal University, 420008 Kazan, Russia; 2 Institute of Cytology, Russian Academy of Sciences, 199034 St. Petersburg, Russia; ranaesc@gmail.com (A.S.); litvinchukspartak@yandex.ru (S.L.); 3 Samara Federal Research Center of the Russian Academy of Sciences, Institute of Ecology of Volga Basin, Russian Academy of Sciences, 445003 Tolyatti, Russia; 4 Department of Zoology and Ecology, Penza State University, 440026 Penza, Russia; oaermakov@list.ru; 5 Department of Biology, Genetics and Animal Breeding, Kazan State Academy of Veterinary Medicine Named after N.E. Bauman, 420029 Kazan, Russia; 9949m@mail.ru

**Keywords:** *Pelophylax*, age structure, maximum lifespan, Middle Volga River region, morphologically cryptic forms

## Abstract

An analysis of the age structure of three water frog species (*P*. *lessonae*, *P*. *ridibundus*, *P*. *esculentus*) in the Middle Volga region of Russia was conducted. The skeletochronological method determined their maximum lifespans to be 7, 6, and 4 years, respectively. This study revealed specific characteristics of the age structure of *Pelophylax* frogs in relation to their taxonomic composition when cohabiting (population system type) and the degree of habitat transformation. Differences in body size and lifespan were observed among cryptic forms exhibiting mitochondrial DNA replacement and the presence of various types of heterozygous and homozygous alleles. Water frogs of the genus *Pelophylax* have a complex taxonomic structure, including hybrid species and morphologically cryptic forms, as well as massive mitochondrial DNA introgression. The aim of our paper was to analyze the age structure of three species of water frogs (*P. lessonae*, *P. ridibundus*, and *P. esculentus*) in the Middle Volga River region (European Russia). Based on the skeletochronological method, we determined their maximum lifespans of seven, six, and four years, respectively. The onset of sexual maturity in the marsh frogs occurred after two and four winterings in males and females (respectively), in the pond frog—one to two years, and in the edible frog—one to three years. This study revealed specific characteristics of the age structure of *Pelophylax* frogs in relation to their taxonomic composition when cohabiting (population system type) and the degree of habitat transformation. Differences in body size and lifespan were observed among cryptic forms exhibiting mitochondrial DNA replacement and the presence of various types of heterozygous and homozygous alleles.

## Simple Summary

The age structure of amphibian populations is considered a crucial parameter influencing population dynamics, stability over time, reproductive success, and overall abundance. The age structure is significantly affected by environmental conditions and predator pressure, but it may also be influenced by internal genetic factors. The genus *Pelophylax* shows a complex taxonomic structure, encompassing hybridogenetic and cryptic species. The water frogs display various types of mitochondrial and nuclear DNA, including forms with invasive haplotypes that are prevalent in the northern and northeastern parts of their ranges. Herein, an analysis of the age structure of water frog populations with different genetic structures were presented.

## 1. Introduction

The age structure is one of the most important characteristics of animal populations. Poikilothermic vertebrates grow throughout their lives, which makes it somewhat easier to determine their age. In the past, the lifespan of amphibians was determined primarily based on their size [1]. The inaccuracy of this method, due to the heterogeneity of populations (size ranges for various age groups can overlap significantly), makes this method insufficiently informative. A more accurate method for determining the age of vertebrates is to tag and then recapture them [2,3]. The disadvantage of this method is its high labor intensity and the need for long-term observations. The skeletochronological method is most widely used in zoological practice due to its relative simplicity [4,5,6]. This method is based on counting the cyclical lines of arrested growth (LAG) in long tubular bones, which are formed because of growth inhibition during hibernation [6,7,8,9].

Water frogs of the genus *Pelophylax* Fitzinger, 1843 have become a common subject for age structure studies due to their wide distribution (e.g., [10,11,12]). Therefore, a comparative study of the age structure of populations of water frogs from different parts of their ranges is of great interest. Previously, a number of studies were conducted on tagging [2,13] and skeletochronological studies [14,15,16,17] of populations of water frogs in the Middle Volga River region. In addition to these frogs, skeletochronological studies were also performed in this region on toads of the genera *Bufo* Garsault, 1764 and *Bufotes* Rafinesque, 1815 [15,18].

In the Middle Volga River region, water frogs of the *P. esculentus* complex inhabit the eastern part of their ranges [19]. The complex includes two parental species (the marsh frog *P. ridibundus* (Pallas, 1771) and the pond frog *P. lessonae* (Camerano, 1882)), and the edible frog *P. esculentus* (Linnaeus, 1758), a form of hybrid origin [20,21]. The hemiclonal reproduction type, with the elimination of a parental genome, usually results in *P. esculentus* coexisting with parental species in so-called “population systems”. However, *P. esculentus* can sometimes live outside the syntopic zone with the parental species and even form its own separate populations (pure E-type population systems involving triploid individuals) [19,22,23,24].

It is also important to note that in Eastern Europe, *P. ridibundus* is represented by a complex of morphologically cryptic forms, of which “western” and “eastern” forms are usually distinguished, which some authors consider to be separate species, respectively, *P. ridibundus* and *P.* cf. *bedriagae* [25,26,27,28,29,30,31]. In addition, mitochondrial (mtDNA) haplotypes of the Balkan form *P. kurtmuelleri* (Gayda, 1940) are occasionally found here [30,31]. Environmental modeling revealed significant differences in habitat preferences between *P. ridibundus* and *P.* cf. *bedriagae* [31]. Thus, identifying differences in the survival strategies of various species of water frogs living together and forming mixed population systems is a very relevant and modern problem [32]. The age structure of these species in the Middle Volga River region requires additional analysis, given the very complex taxonomic composition of this complex here. Therefore, the aim of our paper was to study the age structure of local water frog populations, taking into account genetically confirmed data on the hybridogenic species *P. esculentus*, as well as eastern and western forms of *P. ridibundus*.

## 2. Material and Methods

### 2.1. Description of the Area and Object of Research

**Figure 1:**
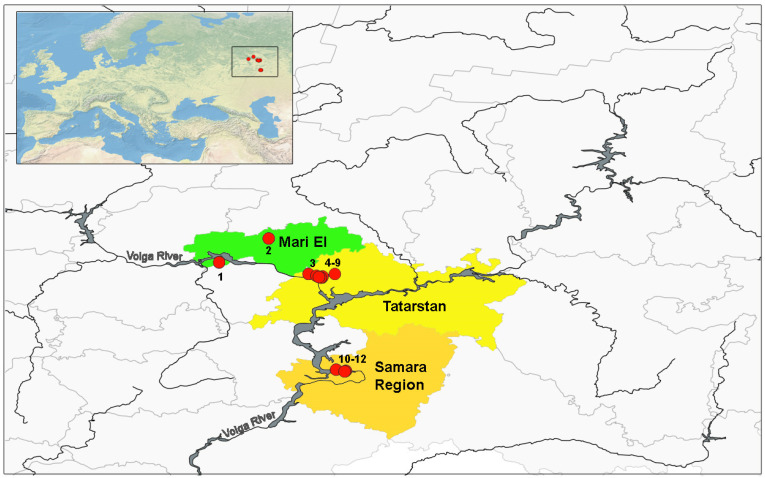
Map of the Middle Volga River region (Russia) with studied localities of water frogs of the genus *Pelophylax*. The inset of the map indicates localities studied on the European map. The numbers of localities correspond to those indicated in Table 1.

We studied the age structure of water frogs in three administrative regions of Russia: Samarskaya oblast’, republics of Mari El and Tatarstan (Figure 1; Table 1). We collected samples of three species of water frogs with a hand net in water bodies where adult individuals reproduced and tadpoles developed before metamorphosis. For analysis, we selected populations inhabiting permanent stagnant reservoirs with varying degrees of landscape transformation and human development of the adjacent territory. Depending on the dominant type of development, the studied territories were divided into five zones [14]: Z1—industrial zone (territory adjacent to industrial facilities, wastelands, septic tanks, industrial and production waste dumps); Z2—high-rise development zone, including historical urban centers (other technogenic territories); Z3—low-rise development zone, as well as adjacent parks located within cities and summer cottage areas (the anthropogenic impact is expressed in insignificant transformation of natural landscapes and little pollution); Z4—forest park zone, these are urban and suburban forest parks; ZC5—control zone, these are territories remote from cities with minimal anthropogenic transformation; as a rule, these are protected natural territories such as nature reserves and sanctuaries.

It should be noted that our studies of water frog population systems were conducted in areas with various levels of habitat transformation, including in such large cities as Kazan and Togliatti.

The primary identification of species was carried out via analysis of standard morphological characteristics [30]. Specimens that are difficult to identify based on external morphological traits (*P. esculentus*, *P. ridibundus* and *P.* cf. *bedriagae*) were identified using DNA flow cytometry [33] and molecular genetic methods [30]. In studying genetic variability, two standard markers were used: a fragment of the first subunit of the cytochrome oxidase mitochondrial gene (COI) and a fragment of intron 1 of the serum albumin nuclear gene (SAI-1) [29,30,34]. Statistical processing was carried out using Statistica 8.0.

The body length (SVL) of each individual was measured using digital caliper with an accuracy of 0.01 mm.

### 2.2. Study of Age Structure in Water Frogs

Age was determined using the skeletochronological method [18]. The middle of the diaphysis of the second phalanx of the fourth finger of the right hind limb (cross sections were 23 μm thick) was taken for the analysis. This study was carried out using a microtome-cryostat MK-25. Sectioning and staining were performed using standard methods [15]. The middle of the diaphysis of the second phalanx of the fourth finger of the right hind limb was taken for the analysis. Before making cross sections, we performed a decalcification procedure in a 2–3% HNO_3_ solution. Cross sections of 23 µm thickness were prepared using standard techniques. We performed staining with Ehrlich’s acid hematoxylin. We placed the prepared preparations in a clean glycerin medium and covered them with a cover glass for better preservation. To determine the rate of bone resorption from the endosteum, the diameter (D) of the bone marrow cavity and diameters (the average value of the sum of the maximum and minimum cross-sectional diameters) of the irregular circles formed by the first, second, and subsequent LAGs were compared (Table S1). We measured the diameter of the medullary cavity based on the diameter of the endosteal ring, since the resorption of the primarily deposited periosteal bone, in which LAGs are formed, occurs from the endosteal side, while the medullary cavity expands and a layer of endosteal bone is formed. In order to ensure that the comparison of the above measurements was correct, we selected sections from a strictly defined area, where the opening of the blood vessel passes. This opening is located in the center of the diaphysis of tubular bones, where the periosteal layer of the bone is widest. Measurements were taken using an ocular micrometer (division value was 0.01 mm). The use of tubular bones for age determination in amphibians is preferable to flat bones due to better visibility of the layered structure. However, the use of tubular bones as a recording structure is associated with a possible error associated with the resorption of LAGs in young animals before sexual maturity. It is generally accepted that after the start of sexual maturity, the rate of resorption of LAGs corresponding to the first winterings from the endosteal cavity side slows down sharply and can be neglected [35,36,37,38,39,40,41]. The obtained preparations were fixed in glycerol. Sections were measured using an object-micrometer (OmO, 0.01 mm) and an ocular micrometer, as well as a USB camera (DCM310E, USB 2.0, 3 Mpixels, Scopetek, Moscow, Russia). 

**Figure 2:**
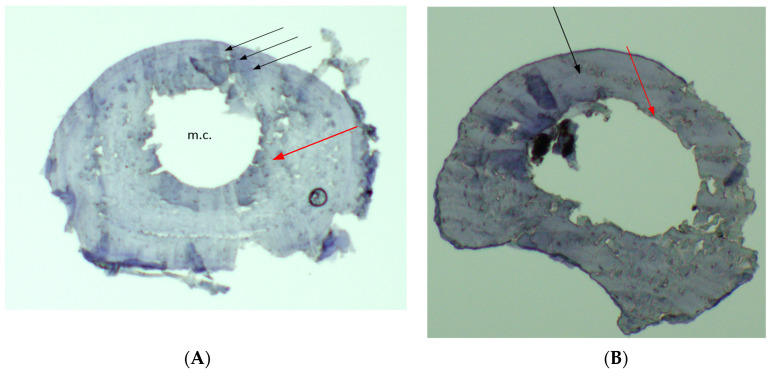
Cross sections obtained from diaphyses of phalanges of water frogs. (**A**) 5-year-old individual (female, SVL = 95.16 mm); (**B**) 3-year-old individual (male, SVL = 61.07 mm). Arrows show lines of arrested growth (LAGs). The innermost red arrow points to the resorption line.

During life, the increase in bone thickness occurs due to the periosteum. Most species of Northern Eurasian amphibians lack true osteons in their bones. Therefore, internal restructuring of bone tissue and the associated intercalary resorption almost do not occur in them [36]. LAGs in flat bones persist throughout life. In long bones with an internal cavity, resorption processes occur from the endosteum, which can cause the first layers to partially or completely disappear (Figure 2). The rate of resorption varies among species and is not constant throughout the life of an individual. After reaching sexual maturity, due to the general slowdown in bone growth, resorption processes decrease or stop completely. LAGs that have not resolved by the start of maturity, as well as those that form after it, persist throughout life.

Therefore, to correctly determine age, we counted the number of LAGs that had time to disappear before the animal reached sexual maturity. To do this, we compared the transverse dimensions of the bones of juveniles and one-year-old individuals with the dimensions of the bone marrow cavity and with the diameter of the bone limited by the first fully visible LAG, and, if necessary, by subsequent LAGs in subadult and adult animals [8,35,36,37,38,39,40,41]. In adult frogs, we also estimated the relative mass of the gonads.

For comparisons of two samples, the Mann–Whitney U test and the Kruskal–Wallis test were used. To identify the relationships between parameters, Spearman’s rank correlation coefficient was performed. All calculations were performed using Statistica StatSoft 8.0 software.

## 3. Results

**Figure 3:**
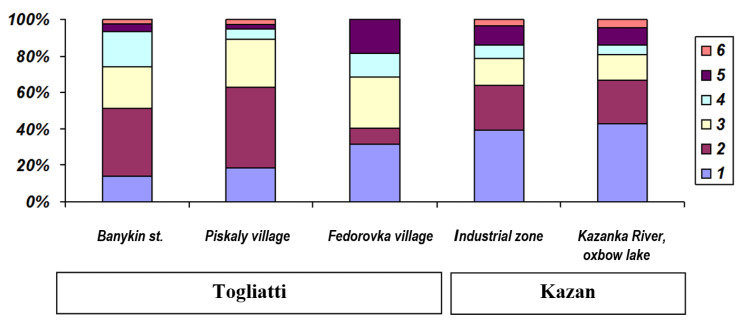
Age structure of populations of the *Pelophylax ridibundus* Togliatti: green zone (forest park in Banykino st.), low-rise development (Piskaly village), control by development (Fedorovka village); Kazan: industrial zone (gunpowder plant), control by development (oxbow lake). The age of adult and immature frogs is indicated in the rectangle on the right.

**Figure 4:**
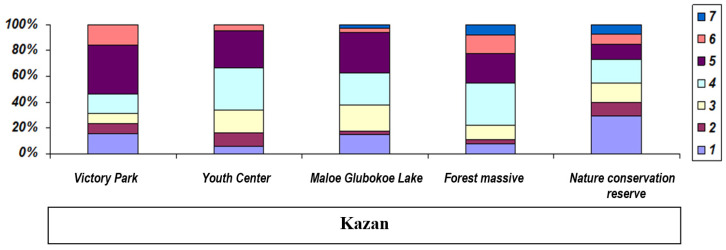
Age structure of populations of *Pelophylax lessonae*: Victory Park (residential development—park in residential development); Youth Center (multi-storey residential building); Maloe Glubokoe Lake (suburban forest park); Forest massive (control); Nature conservation reserve (control, nature reserve). The age of adult and immature frogs is indicated in the rectangle on the right.

**Figure 5:**
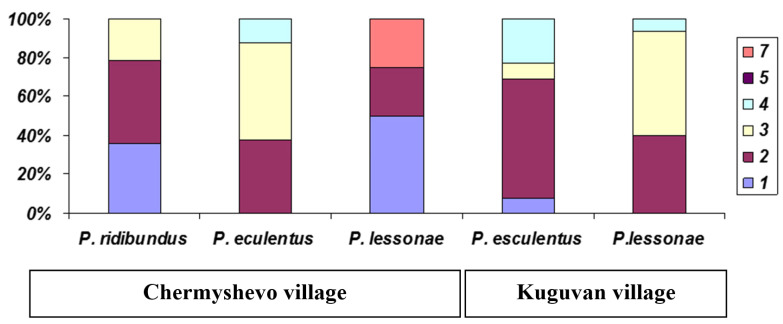
Age structure of various species of water frogs in mixed-population systems (Republic of Mari El). The age of adult and immature frogs is indicated in the rectangle on the right.

The age structure of various species of water frogs in the Middle Volga River region is shown in Table 1. In *P. ridibundus*, the maximum lifespan decreased to the northwest (length of a transect studied was about 350 km): from six years for both sexes in the Samarskaya oblast’ (localities 10 and 11; Table 1, Figure 3) to five years for males in the Republic of Tatarstan (8–9) and three years in the Republic of Mari El (1). The maximum age of male pond frogs was four years in the north-western part the Republic of Mari El «Kuguvan village» (locality 2 in Table 1; Figure 4), which was clearly lower than in the more south-eastern Republic of Tatarstan (seven years). The maximum lifespan of *P. esculentus* did not exceed four years in any of the studied localities (Figure 5).

At the same time, we revealed some differences in the age structure of water frogs depending on the degree of habitat anthropogenic transformation. In heavily transformed landscapes in Kazan City (Youth Center and Victory Park), the maximum lifespan of *P. lessonae* did not exceed six years, whereas on the outskirts of the urban area, it reached seven years, which is comparable with the control territories outside large cities. In the transformed landscapes of Kazan, the maximum age of males did not exceed five years (the “oxbow lake of the Kazanka River”; locality 8 in Table 1), as was the case with females in Tolyatti.

The age of individuals collected at breeding sites (presumably sexually mature) ranged from one to seven years for females and from one to five years for males of all three water frog species. The mean age of females and males did not differ statistically significantly in all three species (*P. ridibundus*: Mann–Whitney test, U = 33.5, *p* = 0.882; *P. lessonae*: U = 91.5, *p* = 0.720; *P. esculentus*: U = 42.5; *p* = 0.200). When comparing the mean age of various taxa, differences were found between *P. ridibundus* and *P. esculentus* (Kruskal–Wallis test, H = 9.61; *p* = 0.008). In the pond frog, individuals older than four years (8.7%) were found only from the vicinity of Kuguvan village (locality 2, Table 1, Figure 5). Individuals with a body length of less than 50 mm sometimes did not have a resorbed gluing line corresponding to the first wintering. However, since frogs were collected before the emergence of yearlings, they were assigned to the age group 1+. A highly statistically significant correlation was found between body length and age of frogs (*P. ridibundus*: R = 0.719, *p* < 0.05; *P. lessonae*: R = 0.776, *p* < 0.05; *P. esculentus*: R = 0.602, *p* < 0.05). However, the oldest individuals were not always the largest. Thus, the 7+ year old *P. lessonae* female had a body size of 77 mm and was actually the largest of the species, but the 5+ year old female had a body size of 70 mm and was smaller in size than two three-year-old individuals.

In the lake frog, age and taxonomic composition were analyzed in two morphologically cryptic species using molecular genetic markers (Table 2). We found a relationship between the presence of alleles of the western form and the age of the studied individuals. In individuals of both sexes that had only alleles of this form, both the average and maximum ages were the greatest. In heterozygous individuals (alleles RB with haplotypes B), these ages were somewhat lower, and among other heterozygous individuals and individuals with alleles of the eastern form, we found only yearlings.

### Determination of the Age of Start of Sexual Maturity

The minimum age of sexual maturity for *P. ridibundus* was two years, since we captured individuals after the onset of the breeding season. For males and most females, the maximum age at which sexual maturity occurred was three years (wintering). In a few females, given their size (SVL = 49.61 mm) and relative ovarian mass, sexual maturity occurred at four years. In *P. lessonae*, this age for most individuals was two years (minimum one year).

**Figure 6:**
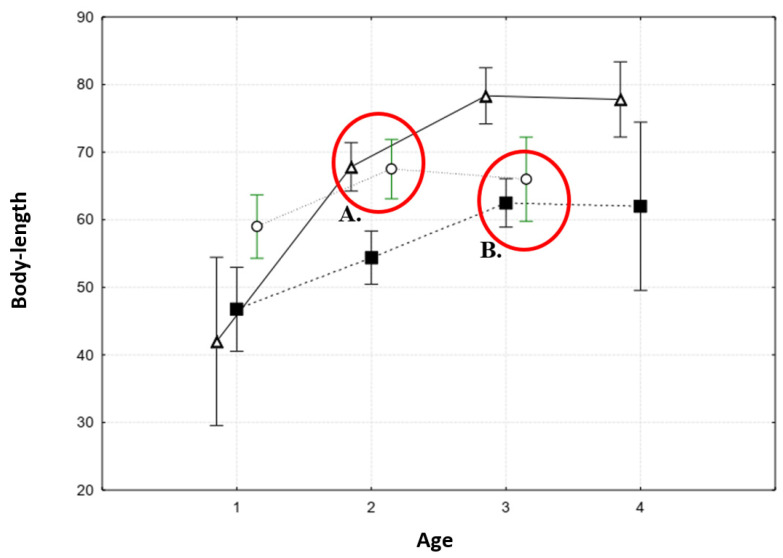
Age-to-body-length relationship in tree species of water frogs from mixed population systems (localities 1 and 2, Table 1). Circle is *Pelophylax ridibundus*, triangular is *P. esculentus*, and square is *P. lessonae*. The interval corresponds to the standard deviation. The red circles show two size–age cohorts: A. is *P. ridibundus* and *P. esculentus*, and B. is *P. ridibundus* and *P. lessonae*.

Particular attention should be paid to the timing of sexual maturity of water frogs in mixed population systems. The onset of sexual maturity in most individuals of each species was clearly visible based on a change in body length growth (Figure 6). After the start of sexual maturity, the growth rate slowed down significantly. In *P. ridibundus*, this occurred at the age of two years, and in the other two species, it occurred at the age of three years. In addition, among the studied species, two size-age cohorts could be clearly distinguished. *P. esculentus* and *P. ridibundus* had approximately the same average length at two years of age, and *P. lessonae* and *P. ridibundus* s had the same average length at three years of age. It should also be noted that in both mixed population systems of Republic of Mari El (localities 1 and 2, Table 1), individuals with an average age of two–three years predominated.

## 4. Discussion

A comparison of our data with the literature showed that the maximum age of water frogs (with the exception of the *P. lessonae*) is generally similar to that in neighboring regions but is clearly lower than in more southern populations [3,10,11,12,32,42,43,44,45,46,47,48,49,50,51,52,53,54,55,56,57,58,59,60,61,62,63,64,65,66,67,68,69,70,71,72,73,74,75] (Table 3). For the *P. lessonae* in the Middle Volga River region, the maximum lifespan was previously estimated at 9 years (wintering) for females and 12 years for males. These data were obtained both based on the method of tagging and recapture [10,42] and the results of skeletochronological analysis [44]. These estimates are 5–6 years higher than those of most populations studied by us and other authors (as a rule, 5–8 years).

*P. ridibundus*, which lives in more southern regions (Turkey and Iran), has a maximum lifespan of 12–13 years [68,72,76], which is significantly longer than that in the region we studied (5–6 years, Table 1 and Table 3). However, it is important to note that other genetic lineages and species of *P. ridibundus* mainly populate these southern regions [25,31]. The maximum age of the hybridogenous *P. esculentus* in the south of its range (Romania) reaches 10 years for both females and males [75], which is significantly greater than in the Middle Volga River region (4 years, Table 1).

Among the other species of this genus, long lifespans have been recorded for *P. caralitanus* (Arikan, 1988) [77] from Turkey—10 years for females and 9 years for males according to [78,79]—and *P. saharicus* (Boulenger, 1913) from Morocco [80]. A significantly shorter maximum lifespan (5–6 years) was noted in *P. persicus* (Schneider, 1799) from Tajikistan [81], *P. epeiroticus* (Schneider, Sofianidou & Kyriakopoulou-Sklavounou, 1984) [82] from Greece [83], and *P. perezi* (López-Seoane, 1885) from Spain [84,85].

*Pelophylax nigromaculatus* (Hallowell, 1861) is characterized by a relatively short lifespan (5–6 years for females and 4–6 years for males in China and Japan, respectively) [86,87]. In South Korea, males of this species were found to be up to 8 years of age, with an average age of 4.4 years [88].

It should be noted that in mixed-population systems, competition among breeding males could have a significant impact on their age composition. Pairs of male *P. esculentus* with large female *P. lessonae* usually dominate in amplexus, while pairs between females and males of *P. esculentus* are extremely rare, if observed at all [89]. Similar pairs in amplexus can be observed in populations involving triploid individuals of *P. esculentus*. In this case, a shorter maximum lifespan of triploid individuals was noted compared to diploid individuals of the hybridogenic species [32,52]. The role of competition is also noted, manifested in the exclusion of young sexually mature males (before the third wintering) from participation in mating. It is believed that young males are more likely to be excluded from mating due to competition from older males [54]. At the same time, observed differences in the average and maximum lifespan in mixed-population systems may also reflect transformation processes, including low abundance of one of the parental species (e.g., the pond frog) in the reproductive part of the population. It should be noted that a decrease in the number of this species in mixed populations in various parts of its range has been observed [90,91].

**Figure 7:**
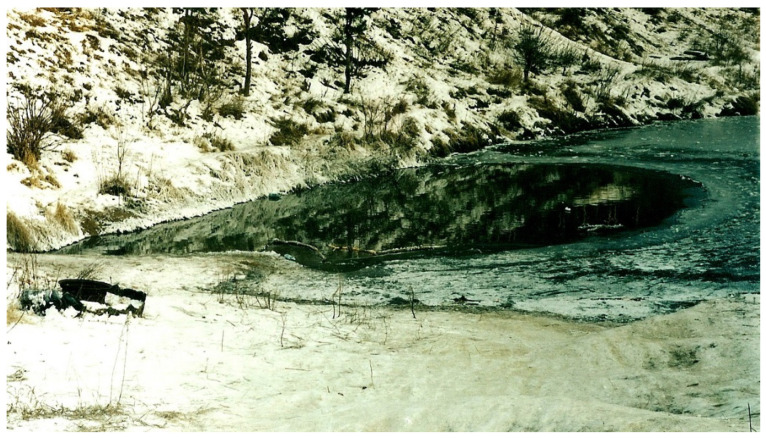
Ice-free winter washout in lake near Banykino street in Samarskaya oblast’ caused by thermal pollution.

Two morphologically cryptic forms of the *P. ridibundus* inhabit the study region [29,30,31,92]. According to our data (Table 2), the maximum and average ages were observed in forms with the presence of alleles of the western form and some heterozygous genotypes, but individuals with alleles of the eastern form were not found among sexually mature individuals. This is probably due to the fact that in Samarskaya oblast’, the frequency of alleles of the western form was significantly higher than that of the eastern one [27,31]. *P. ridibundus* with alleles of the western form and eastern mtDNA are characteristic of the geographic and ecological periphery of the species’ range and anthropologically transformed habitats [27]. According to our data (Table 2), the maximum size (SVL = 108.9 mm) was found in a female of the western form with mitochondrial DNA replacement and in a male (SVL = 91.6 mm) with heterozygous SAI-I alleles and mitochondrial DNA of the eastern form. Our data indicate differences in age composition, which may be associated with both the different contributions of each of these cryptic forms to the genotype and with the geographical location of populations, as well as with the degree of anthropogenic transformation of their habitats. In urban conditions, the most important factor is the thermal pollution of urban water bodies associated with storm sewers and wastewater discharge. Therefore, changes in age composition can serve as an indicator of the degree of anthropogenic transformation of landscapes [16], as well as thermal pollution, especially in invasive populations of *P. ridibundus* [55,60]. For example, in reference [60] it is noted that obvious differences in the sizes of adults (and possibly in the age structure) may be influenced by the origin of introduced populations, since one of the studied populations «Refta» (Reftinskoye Reservoir, Sverdlovsk Region) originated from Odesskaya oblast’ (Ukraine) and the second (Verkhniy Tagil) from Krasnodarskiy kray (Caucasus, Russia). These populations are characterized by different frequencies of alleles and haplotypes of western and eastern forms [31,92]. Increased heterozygosity for SAI-I alleles was also noted for thermal sources of the Kamchatka Peninsula [93], where the maximum age was nine years for males and six years for females, with an average age of 3.5 and 3.3 years, respectively [55]. In a reservoir with thermal pollution (Figure 7) in Banykino street in Samarskaya oblast’ (locality 10, Table 1), the longest lifespan (males) was noted in the population, which was characterized by an unusually strong predominance of alleles and haplotypes of the eastern form of *P. ridibundus*.

The age at which water frogs reach sexual maturity is influenced by habitat conditions [94], including growth characteristics in various years [32]. A relationship was noted between the body sizes of individuals of the same age and the onset of sexual maturity and the beginning of participation in reproduction, which was established by studying the dynamics of growth of individuals during life and the slowdown in growth, especially in females [32].

In northern Greece, *P. ridibundus* becomes sexually mature after their first wintering, having an SVL = 62.0–66.0 мм [95]. At the same time, in high-mountain reservoirs of Armenia, only a small part of the population begins to reproduce after the third wintering, and the majority begin after the fourth [65]. In the warmed Reftinsky Reservoir (the Ural Mountains), some females begin to reproduce after the second wintering at a body length of 78.0–83.0 mm, and after the third wintering, all females become sexually mature [58]. In warm water bodies of the Kamchatka Peninsula, sexual maturity in most individuals can occur after the first or second wintering [96]. For the Moscow vicinities (Zvenigorod District), females and males of the *P. ridibundus* reproduce for the first time after the third wintering [96].

It should be noted that for more southern populations of water frogs, stable achievement of sexual maturity occurs in the 3rd–4th year, while the transition to spawning in most cases occurs much later [32]. Here, size groups with different periods of sexual maturation were revealed. Among the relatively smaller individuals, growth retardation in females occurred in the 4th year of life, and in males, this occurred in the 5th and 6th years. Among the relatively larger individuals, growth retardation in females occurred in the 6th year of life, and in males in the 5th, which was confirmed by the analysis of individuals participating in reproduction [32].

## 5. Conclusions

In our study, the age structure of three species of water frogs (*P. lessonae*, *P. ridibundus*, *P. esculentus*) in the Middle Volga River region was studied. The skeletochronological method determined maximum lifespans of 7, 6, and 4 years, respectively. In almost all populations of water frogs, the average age of males was higher than that of females. The average age is affected by the transformation of the habitat within cities (Kazan and Togliatti), as well as thermal pollution of water bodies within these cities. The age structure is influenced by the existence of *P. esculentus* in population systems with two or one parental species. Thus, the parental *P. ridibundus* and *P. lessonae* had the shortest average lifespan in mixed populations containing all three species, while in mixed populations containing *P. lessonae* and *P. esculentus*, the average lifespan of *P. esculentus* was shorter. *P. ridibundus* in the study area was represented by two morphologically cryptic forms, which obviously had different adaptive capabilities. Moreover, the maximum body size was found in a female of the western form with mitochondrial DNA replacement and in a male with heterozygous alleles and mitochondrial DNA of the eastern form. The start of sexual maturity in the *P. ridibundus* occurred after 2–3 winterings in males and up to 4 years in females. In *P. lessonae*, the age of sexual maturity for most individuals was two years, and the minimum was one year. In *P. esculentus*, the average time of reaching sexual maturity was three years.

In the area of this study, the authors conducted an assessment of trophic pressure by analyzing the diet of a typical predator, the common grass snake *Natrix natrix* (Linnaeus, 1758), observing aquatic birds and mammals, and examining the level of invasion by metacercariae of trematodes that complete their development in definitive hosts. Preliminary evaluations of trophic pressure indicated similar or reduced levels under conditions of anthropogenic transformation. There was a comparable composition of predator consumer spectra [97] and levels of metacercarial invasion by trematodes [98,99]. However, under transformed conditions, a reduction in consumer composition was noted due to disturbance factors, particularly in recreational areas, as well as a low level of invasion or a decrease in the species composition of trematode metacercariae [99,100,101,102,103,104,105,106]. Throughout the study area, the composition of green frog consumers is homogeneous in taxonomic composition [13]. For the vast majority of predators, immature individuals are easier prey, while larger and, accordingly, older individuals experience less pressure. On the other hand, in urban conditions, the trophic load decreases (due to the disturbance factor), and the composition and density of natural predators in control areas, on the contrary, is higher. According to our observations and literary data, synanthropic predators in the study area generally do not consume large aquatic amphibians. The main predator of water frogs is the grass snake (*Natrix natrix*), as well as waterbirds. In the control zone, the main additional daytime and nighttime predators are predatory and water-dwelling mammals. An invasive species, the American mink *Neogale vison* (Schreber, 1777), is also common outside populated areas. However, in the localities we surveyed, it was not observed (our data). Indirect data on the pressure of predators were provided based on an analysis of the degree of infection with helminths, in particular metacercariae of trematodes, for which consumers are the final hosts. Under transformation conditions, the degree of infection is usually lower or similar to urban conditions compared to control zones (our data). Therefore, we believe that predator pressure was not a determining factor in the studied populations.

## Figures and Tables

**Table 1:** Species composition, localities, sample size, sex, average (adult and immature individuals), and maximum ages in three species of water frogs inhabiting the Middle Volga River region (Russia). *Pelophylax ridibundus* includes both morphologically cryptic western and eastern forms. Nu is number of localities; N is coordinates of the northern and eastern regions of the eastern longitudes; n is the sample size.

Locality			Coordinates		Species	n	Sex	Average Age	Maximum Lifespan
Nu	Name	Zone	N	E					
**Republic of Mari El**									
1	Chermyshevo village	ZC5	56.183	46.516	P. ridibundus **	14	♀	1.8	3
						5	♂	1.9	3
					P. esculentus *	3	♀	2.3	3
						8	♂	2.8	4
					*P. lessonae*	3	♀	2.0	3
						4	♂	1.3	2
2	Kuguvan village	ZC5	56.783	47.766	P. esculentus *	3	♀	3.3	4
						1	♂	-	4
					*P. lessonae*	8	♀	3.3	7
						15	♂	2.7	4
**Kazan City, Republic of Tatarstan**									
3	Nature conservation reserve	ZC5	55.900	48.752	*P. lessonae*	20	♀	3.6	7
						34	♂	3.3	7
4	Forest massive	ZC5	55.900	49.414	*P. lessonae*	14	♀	4.1	7
						13	♂	3.6	7
5	Maloe Glubokoe Lake	Z4	55.847	48.962	*P. lessonae*	41	♀	3.7	7
						40	♂	3.3	7
6	Youth Center	Z2	55.809	49.100	*P. lessonae*	29	♀	3.4	6
						19	♂	3.2	6
7	Victory Park	Z2	55.833	49.111	*P. lessonae*	11	♀	3.3	6
						15	♂	3.1	6
8	Kazanka River oxbow lake	Z1	55.804	49.068	P. ridibundus **	9	♀	2.9	5
						12	♂	2.3	5
9	Industrial zone	Z1	55.824	49.015	P. ridibundus **	13	♀	2.7	6
						15	♂	2.1	5
**Togliatti City, Samarskaya oblast’**									
10	Banykino st.	Z4	53.500	49.439	P. ridibundus **	25	♀	2.9	5
						18	♂	2.7	6
11	Piskaly village	Z3	53.470	49.690	P. ridibundus **	18	♀	2.1	6
						20	♂	2.6	4
12	Fedorovka village	ZC5	53.466	49.665	*P. ridibundus* **	19	♀	2.9	5
						19	♂	2.4	5

Transformed habitats: Z1—Zone industrial area; Z2—Zone multi-storey buildings; Z3—Zone low-rise buildings; Z4—Zone forest–park zone—urban and suburban forest parks; ZC5—Control zone. Methods of species identification: * DNA flow cytometry; ** molecular genetic analysis.

**Table 2:** Age structure of two morphologically cryptic forms of the *P. ridibundus* frog in the Middle Volga River region based on the results of studying molecular genetic markers. R is alleles and haplotypes of the western form and B of eastern form; n is samples that had different combinations of alleles and haplotypes.

N	SAI-1	COI	n	Sex	Average Age	Maximum Lifespan	Maximum SVL
1	RR	R	2	♂	2.5	3	62.89
			2	♀	4.0	5	82.79
2	RR	B	6	♂	2.3	5	71.99
			4	♀	3.0	5	108.90
3	RB	B	2	♂	2.0	4	91.60
			2	♀	2.5	5	98.70
4	RB	R	1	♀	0+	0+	34.58
5	BB	R	1	♀	0+	0+	33.37

**Table 3:** Age structure of water frog populations of the *Pelophylax esculentus* complex according to literature data. M is tagging and recapture method; S is skeletochronological method.

Country, Province	Maximum Lifespan		Average Age (Adults)		Method	Source
	Males	Females	Males	Females		
*P. lessonae*						
Russia, Ryazanskaya Oblast’	9	12	-		M	[42]
Russia, Republic of Tatarstan (near Kazan)	9		-		M	[43]
	12		-		S	[44]
	5		-		S	[45]
Russia, Republic of Tatarstan (Krugloe Lake)	4		-		S	[45]
Russia, Leningradskaya oblast’ (Luga)	6	6	-		S	[10]
Russia, Ivanovskaya oblast’	7		-		S	[46]
Russia, Ivanovskaya oblast’	7		-		S	[47]
Russia, Ivanovskaya oblast’	4 *		1.93 *		S	[48]
Russia, Ivanovskaya oblast’	8		-		S	[49]
Forest-steppe zone of Ukraine	6		-		M	[3]
Ukraine, Chernovitskaya oblast’	7	8	3.15	3.56	S	[50]
Ukraine, Vinnitskaya oblast’	4	4	2.56	3.17	S	[51]
Ukraine, Khar’kovskaya oblast’	5	7	3.78	5.00	S	[52]
Croatia, Lova River	8		4.80		S	[53]
Poland, Wrocław (Raków)	7	8	-		S	[54]
*P. ridibundus* (including southern lineages)						
Russia, Ivanovskaya oblast’	11 (12)		-		S	[46]
Russia, Ivanovskaya oblast’	5 *		2.57 *		S	[48]
Russia, Ivanovskaya oblast’	7 *		-		S	[48]
Russia, Ivanovskaya oblast’	11		-		S	[47]
Russia, Republic of Tatarstan (near Kazan)	11		-		S	[44]
Russia, Kamchatskaya oblast’	6	9	3.30	3.49	S	[55]
Russia, the Middle Urals	9	-	4.90	-	S	[56]
Russia, Sverdlovskaya oblast’ (Verkhniy Tagil)	6		-		S	[57]
Russia, Sverdlovskaya oblast’ (Refta)	-	8	-		S	[58]
Russia, Sverdlovskaya oblast’ (Verkhniy Tagil)	-	9	-		S	[58]
Russia, Sverdlovskaya oblast’ (Verkhniy Tagil)	10	10	-		S	[59]
Russia, Sverdlovskaya oblast’ (Refta)	11		-		S	[59]
Russia, Sverdlovskaya oblast’ (Nizhniy Tagil)	5	5	-		S	[60]
Forest-steppe zone of Ukraine	7		-		M	[3]
Ukraine, Chernovitskaya oblast’	6	8	4.59	4.06	S	[50]
Ukraine, Chernovitskaya oblast’	7	8	4.57	4.47	S	[61]
Ukraine, Vinnitskaya oblast’	6	6	4.66	5.42	S	[51]
Ukraine, Khar’kovskaya oblast’	7	9	5.42	4.30	S	[52]
Ukraine, Khar’kovskaya oblast’	7	10	5.75	4.36	S	[32]
Ukraine, Khar’kovskaya oblast’	9		4.15		S	[62]
Ukraine, Dnepropetrovskaya oblast’	7		-		S	[63]
Georgia	7		4.03	2.78	S	[64]
Armenia, Sevan Lake	10	9	5.80	5.87	S	[65]
Armenia, Khosrov Nature Reserve	8	9	6.54	8.40	S	[65]
Armenia, Razdan River	5	6	3.67	4.63	S	[65]
Turkey	7	11	4.89	5.32	S	[66]
Turkey (Artvin)	6	6	-	-	S	[12]
Turkey	6	7	3.72	3.90	S	[67]
Turkey	11	13	5.42	6.19	S	[68]
Turkey	7	6	3.72	3.77	S	[69]
Poland	7	6	4.40	3.70	S	[11]
Bulgaria, Rozov Kladenets	3	5	2.16	3.25	S	[70]
Bulgaria, Topolnitsa reservoir	3	4	2.28	2.63	S	[70]
Greece	5	5	3.73	2.96	S	[71]
Iran	12	7	5.40	3.00	S	[72]
Iran	11	7	4.50	6.43	S	[73]
Croatia	13		8.00		S	[53]
*P. esculentus*						
Russia, Ivanovskaya oblast’	4 *		1.22 *		S	[48]
Ukraine, Chernovitskaya oblast’	7	8	4.47	5.19	S	[50]
Ukraine, Vinnitskaya oblast’	4	4	3.47	3.16	S	[51]
Ukraine, Khar’kovskaya oblast’	10		3.63		S	[62]
Ukraine, Khar’kovskaya oblast’	7	10	5.04	4.04	S	[52]
Ukraine, Khar’kovskaya oblast’	8	9	4.83	4.46	S	[32]
Sweden	6	6	-	-	S	[74]
Romania	10	10	6.70	5.00	S	[75]
Croatia	10		5.10		S	[53]

* Authors counted only number of LAGs without correction for bone resorption.

## Data Availability

The data presented in this study are available upon request from the corresponding author.

## Supplementary Materials

The following supporting information can be downloaded at: https://www.mdpi.com/article/10.3390/ani15091273/s1, Table S1: Diameter of the medullary cavity and bone in the middle of the diaphysis of the phalanx of the fourth finger of the right hind limb in water frogs.
