# Supplementary material for: Age Structure of Water Frogs of the Genus Pelophylax in the Middle Volga River Region (European Russia)
Source: Animals (Basel). 2025 Apr 30;15(9):1273. doi: 10.3390/ani15091273 (PMC12070855; doi:10.3390/ani15091273)
Supplement: Supplementary file 1 [file animals-15-01273-s001.zip › animals-3590824-supplementary.pdf]

Table S.1. Supplementary materials.

Diameter of the medullary cavity and bone in the middle of the diaphysis of the phalanx of the fourth finger of the right hind limb in water frogs

Locality[a]	Sex	Mean ± standard error	D[b]* Diameter of the medullary cavity in adults	D* of bony, limited				D bone diameter in individuals over 5 years old	D[c]* bone diameter in yuveniles	D[d]* bone diameter in yerlings
				1-st LAG	2-nd LAG	3-rd LAG	4-th LAG			
P. lessonae										
3	♂♂	M±m<br>(n)	219,45<br>±5,17<br>(n=16)	242,55<br>±15,4<br>(n=5)	278,52<br>±23,87<br>(n=7)	322,41<br>±24,31<br>(n=2)	–	407,99<br>±8,58<br>(n=5)	151,47<br>±7,26<br>(n=14)	230,67<br>±4,51<br>(n=19)
	♂♂[c]	M±m<br>(n)	164,45<br>±14,85<br>(n=18)	253,55<br>±15,4<br>(n=3)	268,84<br>±13,86<br>(n=7)	–	–	–		
	♀♀	M±m<br>(n)	236,28<br>±11,44<br>(n=22)	251,57<br>±8,03<br>(n=7)	292,27<br>±5,28<br>(n=7)	344,52<br>±11,33<br>(n=2)	–	456,94<br>±38,61<br>(n=6)		
P. ridibundus										
10	♀♀	M±m<br>(n)	187,22<br>±24,64<br>(22)	269,28<br>±51,15<br>(22)	300,08<br>±66,44<br>(10)	321,2<br>±70,51<br>(5)	–	–	–	308,66<br>±16,94<br>(3)
	♂♂	M±m<br>(n)	189,42<br>±18,48<br>(16)	254,54<br>±32,78<br>(16)	312,07<br>±28,27<br>(11)	352<br>±31,13<br>(2)	–	–	–	296,34<br>±50,05<br>(3)
11	♀♀	M±m<br>(n)	197,23<br>±30,91<br>(15)	276,65<br>±54,23<br>(15)	315,37<br>±61,6<br>(7)	348,7<br>±87,67<br>(3)	–	–	–	293,92<br>±39,16(3)
	♂♂	M±m<br>(n)	187,66<br>±19,14<br>(17)	284,79<br>±38,06<br>(18)	332,2<br>±24,2<br>(4)	–	–	–	–	282,37<br>±21,01(4)
12	♀♀	M±m<br>(n)	325,967<br>±18,05<br>(11)	443,67<br>±29,94<br>(11)	548,43<br>±45,63<br>(7)	657,56<br>±44,92<br>(3)	–	–	181,167<br>±7,09<br>(11)	–
	♂♂	M±m<br>(n)	258,23<br>±22,76<br>(15)	304,99<br>±25,75<br>(14)	421,12<br>±59,2<br>(6)	412,38<br>±33,03<br>(3)	382,8<br>(1)			

a. – numbers of localities as in Table 1; b. the measurement value is presented in µm; c. – individuals (males) in which the resorption of LAG ceased after the first wintering (in the second year of life). n is sample size.
